# Transcatheter closure of secundum atrial septal defect using Cocoon septal occluder: immediate and long-term results

**DOI:** 10.1186/s43044-022-00298-2

**Published:** 2022-08-13

**Authors:** Santosh Kumar Sinha, Mahmodullah M. Razi, Najeeb Ullah Sofi, Manoj Kumar Rohit, Umeshwar Pandey, Awadhesh Kumar Sharma, Mohit Sachan, Puneet Aggarwal, Mukesh Jha, Praveen Shukla, Ramesh Thakur, Vinay Krishna, Rakesh Kumar Verma

**Affiliations:** 1 Department of Cardiology, LPS Institute of Cardiology, GSVM, GT Road, Swaroop Nagar, Kanpur, UP 208002 India; 2 Department of Cardiology, Postgraduate Institute of Medical Education and Research, Chandigarh, India; 3 Department of Cardiology, Atal Bihari Vajpayee Institute of Medical Sciences (ABVIMS) and Dr. Ram Manohar Lohia Hospital, New Delhi, India; 4 Department of Cardiology, Sri Aurobindo Institute of Medical Sciences, Indore, India; 5 Department of CVTS, LPS Institute of Cardiology, GSVM, Kanpur, India

**Keywords:** Atrial septal defect, Cocoon septal occluder, Embolization, Transesophageal echo, Transcatheter closure, Amplatzer septal occluder

## Abstract

**Background:**

Atrial septal defect (ASD) is one of the common congenital heart defects. Its management has transformed dramatically in the last 4 decades with the transition from surgical to percutaneous transcatheter closure for most secundum-type ASDs. Various devices are available for transcatheter closure of ASD with Amplatzer atrial septal occluder being most commonly used worldwide. Cocoon septal occlude has a nanocoating of platinum using nano-fusion technology over nitinol framework that imparts better radiopacity and excellent biocompatibility and prevents leaching of nickel into circulation, and by smoothening nitinol wire makes this device very soft and smooth. The aim of this study was to evaluate feasibility, effectiveness, safety, and long-term outcome of transcatheter closure of ASD using Cocoon septal occluder (Vascular Innovation, Thailand).

**Results:**

All patients undergoing transcatheter closure of hemodynamically significant ASD between September 2012 and July 2019 in our institute were included into this single-center, prospective study. Exclusion criteria were defect > 40 mm, unsuitable anatomy, Eisenmenger syndrome, and anomalous pulmonary venous return. Three hundred and twenty patients underwent device closure, of which 238 (74%) were female. The mean age was 14.6 years (range 6–29), and the median weight was 30.2 kg (range 10–53 kg). Procedure was performed under fluoroscopy using transthoracic and transesophageal echocardiography in 298 (93.1%) and 22(6.9%) patients, respectively. Balloon-assisted technique was used, when septal defect was ≥ 34 mm, in 9 (2.8%) patients. The mean diameter of defect and device was 21.4 mm (range 12–36 mm) and 26.9 mm (range 14–40 mm), respectively. Aortic rim was absent in 11 (3.4%) patients. Primary success was achieved in 312 (97.5%) patients. Early embolization to right ventricle was noted in 2 (0.6%) patients. In both cases, 40-mm device was attempted for defect of 36 mm with inadequate aortic rim using balloon-assisted technique. One (0.3%) patient developed perforation of right atrium. All were surgically repaired. Three (0.9%) patients developed complete heart block following device deployment requiring device retrieval. Two patients had had moderate residual shunt at 6 months of follow-up. After mean follow-up of 50.92 months (range 12.5–89 months), no erosion, allergic reactions to nickel, or other major complications were reported.

**Conclusions:**

Percutaneous transcatheter closure of ASD by Cocoon septal occluder (up to 36 mm) is safe and feasible with high success rate and without any significant device-related major complications over long-term follow-up. With unique device design and excellent long-term safety, it could be preferred dual-disk occluder for transcatheter closure of atrial septal defect. In most of the patients, ASD device can be safely deployed under transthoracic echocardiographic guidance.

## Background

An atrial septal defect (ASD), a pre-tricuspid shunt, accounts for approximately 10% of congenital malformation at birth and almost 30% of newly diagnosed malformations. Among four types of ASD, secundum type is most common and accounts for 70% of cases. Normally, untreated ASD gradually increases in size with growing age and only 4% undergo spontaneous closure [1]. The detrimental effect of ASD stems from increased flow through pulmonary bed and volume overload of right-sided chambers which subsequently lead to irreversible pulmonary vascular obstructive disease and myocardial fibrosis. In adult population, effort intolerance, shunt reversal, and death increase with age and reach up to 50% by the end of third decade, which mandates its early closure [2]. Since the first report of transcatheter closure (TCC) of ASD by Mills et al. in the last century, there has been a paradigm shift for therapeutic strategy and has TCC now become a widely accepted alternative to surgery [3, 4]. However, despite its technical simplicity and availability of various new-generation devices and recently introduced Cocoon septal occluder (CSO; Vascular Innovation, Thailand), procedure is still associated with various complications including cardiac erosions and nickel-related allergic reactions. Amplatzer septal occluder (ASO) is the most common device used for ASD closure worldwide. Cocoon septal occluder (CSO) has a nanocoating of platinum using nano-fusion technology over nitinol framework that imparts better radiopacity and excellent biocompatibility and prevents leaching of nickel into circulation, and by smoothening nitinol wire makes this device very soft and smooth. Our aim was to evaluate feasibility, effectiveness, safety, and long-term outcome of transcatheter closure of ASD using Cocoon septal occluder (Vascular Innovation, Thailand).

## Method

### Study design and patient population

A prospective, single-arm, single-center interventional study was conducted from September 2012 to July 2019. A total of 320 patients with ostium secundum ASD underwent transcatheter closure using CSO. Indication for TCC included secundum ASD in patients ≥ 4 years old with (a) echocardiographic evidence of right ventricular (RV) volume overload, (b) significant left to right shunt (Qp/Qs ≥ 1.5/1), and (c) maximum diameter ≤ 40 mm with adequate (≥ 5 mm) inferior rim. Patients having anomalous pulmonary venous connection, associated complex cardiac anomaly, Eisenmenger syndrome, impaired left ventricular systolic function, and inadequate (< 4 mm) inferior vena cava (IVC) rim were excluded. The protocol of study was approved by institutional ethics committee and conformed to principles of good clinical practice and Declaration of Helsinki. Pre-procedural written and informed consent were obtained from all adult patients and parents/legal guardian of minors.

### Device description

**Fig. 1:**
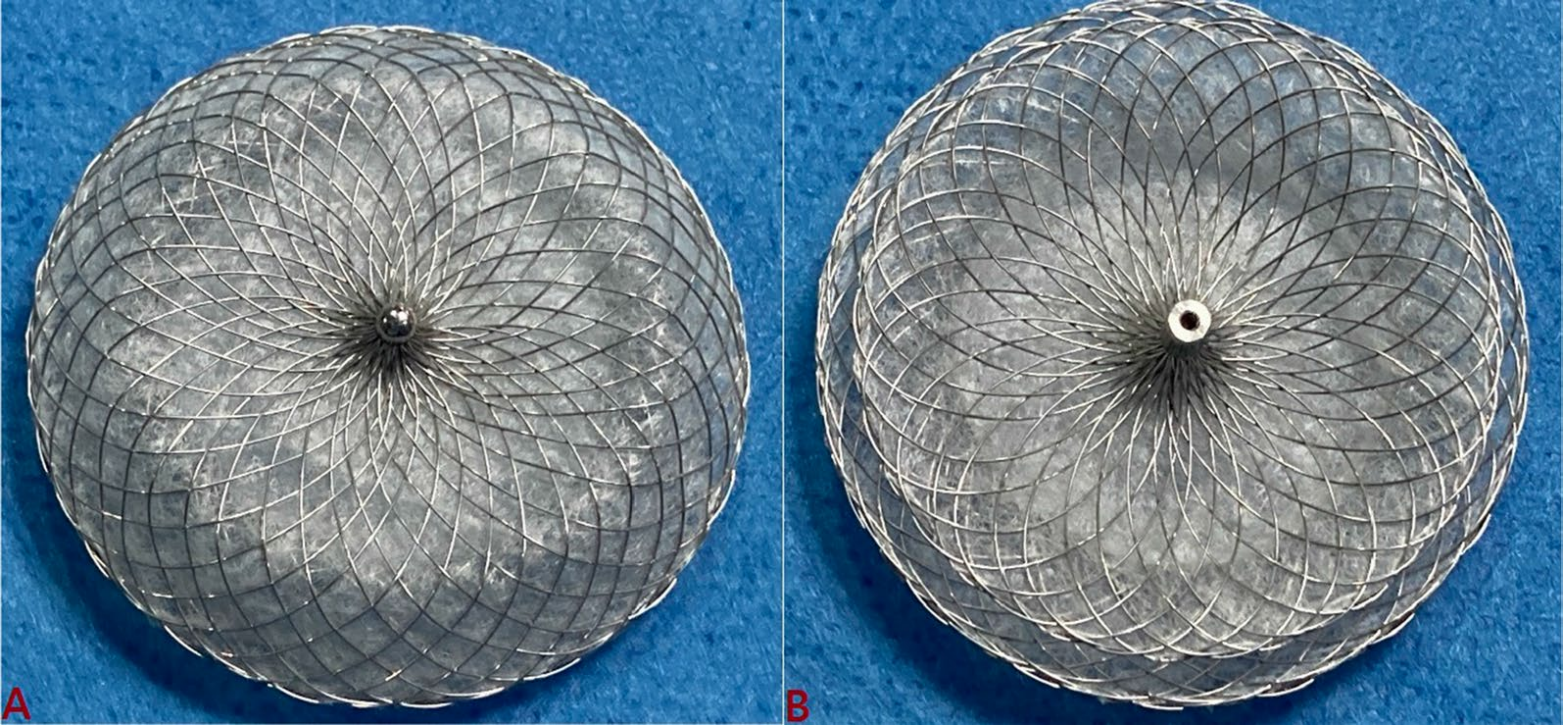
Cocoon septal occluder is a self-expandable, dual-disk structure which is composed of platinum-coated nitinol wire. **A** Left atrial disk; **B** right atrial disk

**Fig. 2:**
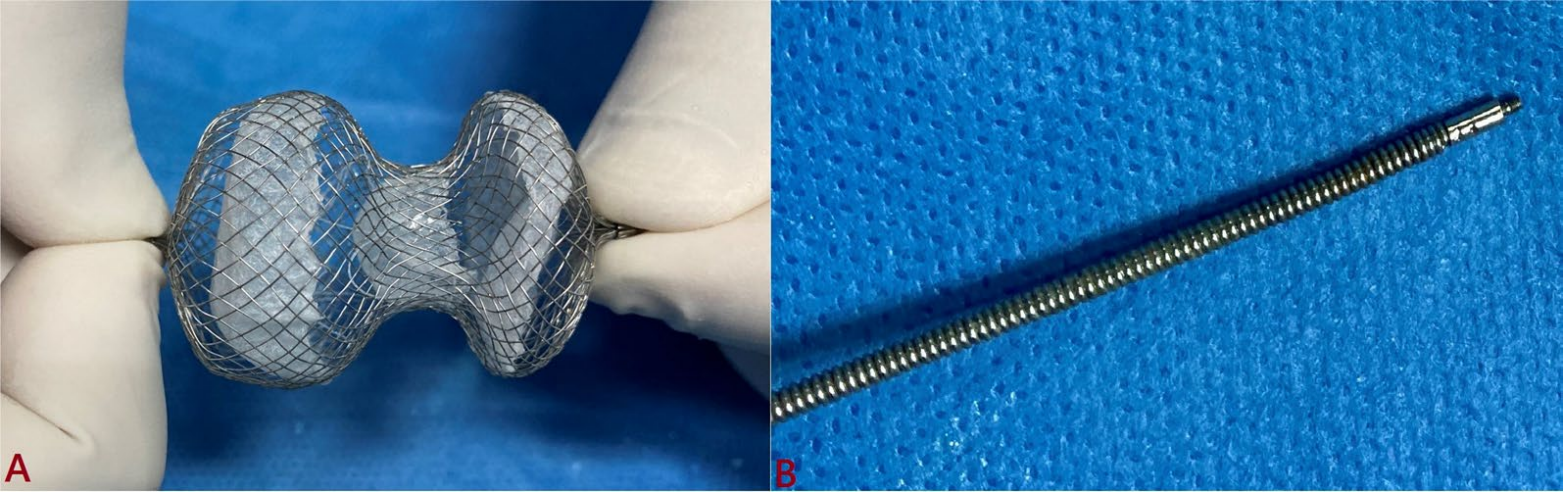
Cocoon septal occlude (CSO) is filled with three polypropylene woven fabrics (**A**); pin vice connector of CSO (**B**)

CSO is a self-expandable, dual-disk structure which is composed of a platinum-coated nitinol wire and is filled with multiple polypropylene woven fabrics (Figs. 1, 2) to facilitate faster and complete closure of defect by inducing thrombogenicity [5]. Nanocoating of platinum using nano-fusion technology imparts better radiopacity and excellent biocompatibility and prevents leaching of nickel into circulation, and by smoothening the nitinol wire makes this device very soft and smooth. Furthermore, it has smaller metal-to-disk ratio, is MRI compatible, and is easy to recapture and reposition during procedure [6]. The waist of device, which is stretchable diameter of ASD, ranges from 8 to 40 mm. The delivery sheath is 80 cm long and angled (90°) with size varying from 8 (for 8–14-mm device) to 14 Fr (for 30–40-mm device). Before introducing device into delivery sheath, it needs to be collapsed into plastic loader by pulling delivery cable.

### Procedural details

#### Crossing the ASD

Right femoral vein was accessed with 5F sheath using modified Seldinger’s technique following which right heart catheterization was performed. The patients were heparinized to keep activated clotting time > 200 s. Ceftriaxone 1 g was administered intravenously. 5F multipurpose catheter (MPA) was advanced over 0.35-inch Terumo wire (Terumo Inc.; Japan) from venous side, and at IVC/right atrium junction it directed toward interatrial septum (IAS) by gently rotating it clockwise in anteroposterior view. Once reached into left atrium (LA), the wire was advanced into left upper pulmonary vein (LUPV) and MPA was parked there. Subsequently, Terumo wire was exchanged with 0.035-inch J-tip super-stiff, exchange length Amplatz wire.

#### Sizing of ASD

**Fig. 3:**
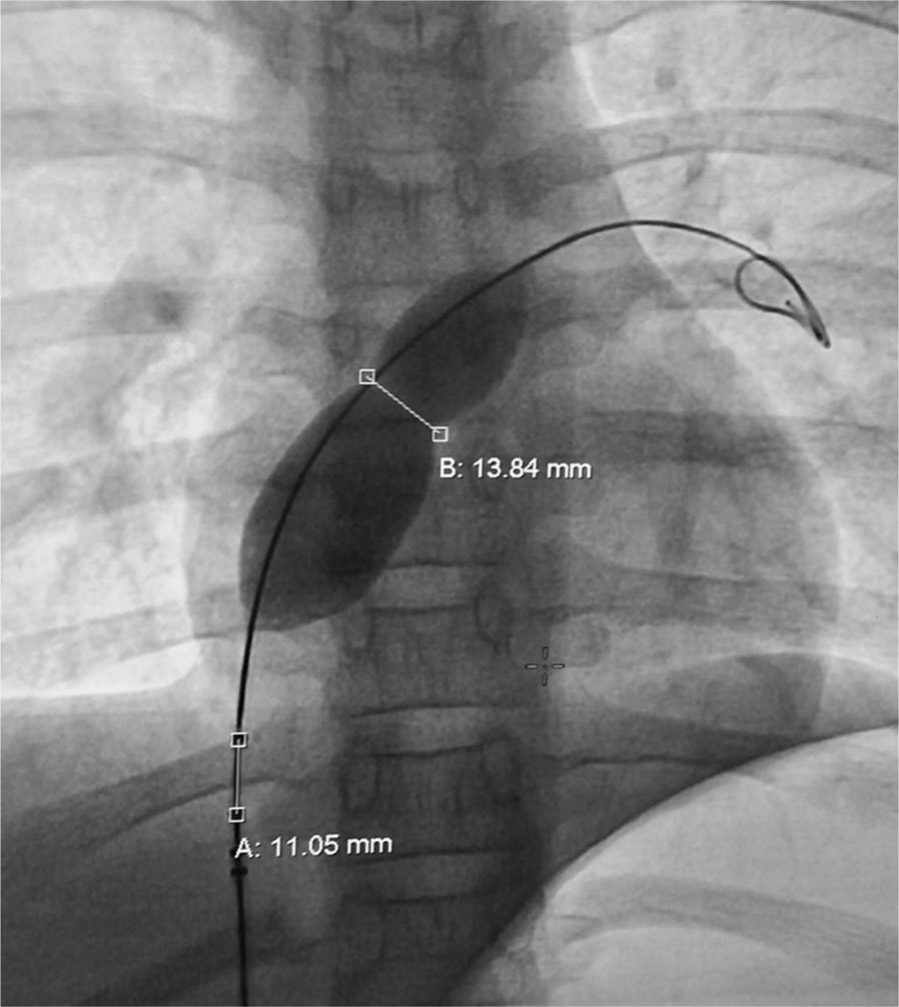
Sizing balloon showing indentation on fluoroscope once inflated with diluted saline contrast after positioning it across the defect and indentation was measured

Procedures were carried out using transthoracic echocardiography (TTE), and transesophageal echocardiography (TEE) was used when TTE was inadequate. ASD was profiled in three planes: apical 4-chamber view (A4C), parasternal short-axis view (PSAX), and bicaval view. A4C showed defect and atrioventricular rim, while PSAX view revealed defect, anterior and posterior rim and bicaval view showed defect, superior and inferior rim. Superior and inferior rim were best profiled using bicaval view, while aortic and posterior rim were best profiled using PSAX view. In defects having flimsy rim and discrepancies between TTE and TEE, sizing balloon (SB; 24 mm/34 mm—Vascular Concept, Thailand) was used. After positioning SB across the defect under echo and fluoro-guidance, it was inflated with diluted contrast until indentation was noted on fluoroscope and flow ceased (stop flow technique–Fig. 3) on echo [7]. In our study, 34-mm balloon was used.

#### Device selection

A device 2–4 mm larger than maximum diameter of defect in patients with adequate rim was used, while it was upsized by 4–8 mm than the largest diameter of defect on BS/TEE/TTE in patients having an inadequate superior/anterior rim. In children with very good transthoracic windows and adequate rim, device ≥ 20% larger than maximum diameter of defect by color flow on TTE was chosen [7].

#### Device deployment

**Fig. 4:**
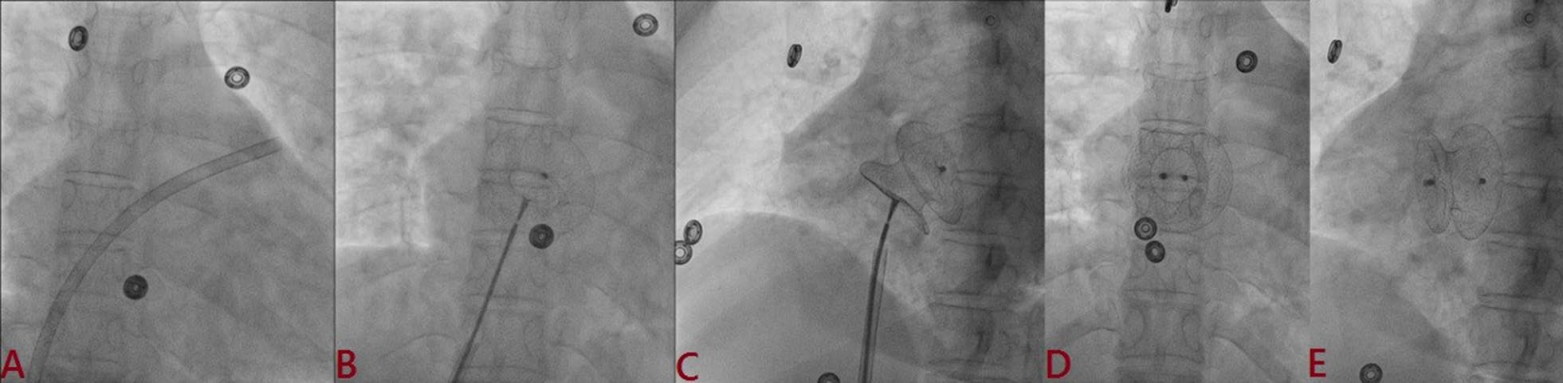
Device deployment using LUPV technique. (**A** Delivery sheath positioned in LUPV; **B** LA disk of device was uncovered and once got snugly fit, RA disk of device was released by gradually pulling the sheath further; **C** Minnesota maneuver was performed to ensure properly sitting disks across defect; **D** device position on AP view; **E** device position on LAO view)

Delivery sheath compatible with device size was advanced over the Amplatz wire till the tip of dilator just reached at outermost margin of cardiac silhouette and dilator and wire were removed, thereby keeping sheath inside LA (Fig. 4A). Device was deployed using left upper pulmonary vein technique [5]. Once device was loaded into sheath and pushed till the tip of sheath, the sheath was gradually pulled into the middle of LA under fluoroscopic guidance and gradually retracted over the cable to open LA disk of the device. The sheath and cable were further pulled. Once LA disk was snugly fitting, the sheath was further pulled to open RA disk of device (Fig. 4B). Minnesota maneuver was performed to ensure properly sitting disks across the ASD (stable position) and its proper position was confirmed using fluoroscope in AP and left anterior oblique (LAO) view (Fig. 4C). In case of repeated prolapse of device into RA despite optimal deployment, it was upsized. Apposition, stability, and any residual shunts were confirmed accordingly using TEE/TTE. Once properly positioned, it was released (Fig. 4D, E).

**Fig. 5:**
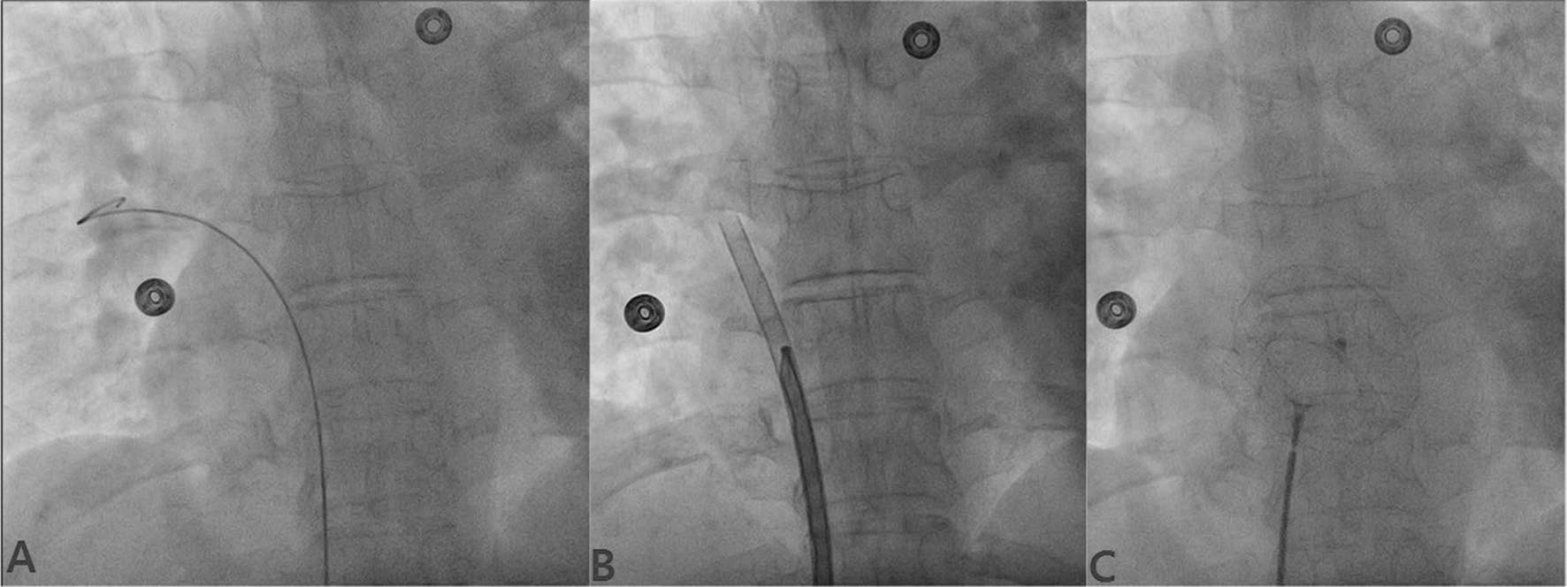
Device deployment using RUPV technique. (**A** Delivery sheath positioned in RUPV; **B** device was loaded inside sheath which was still kept in RUPV; **C** properly positioned disk across the defect)

**Fig. 6:**
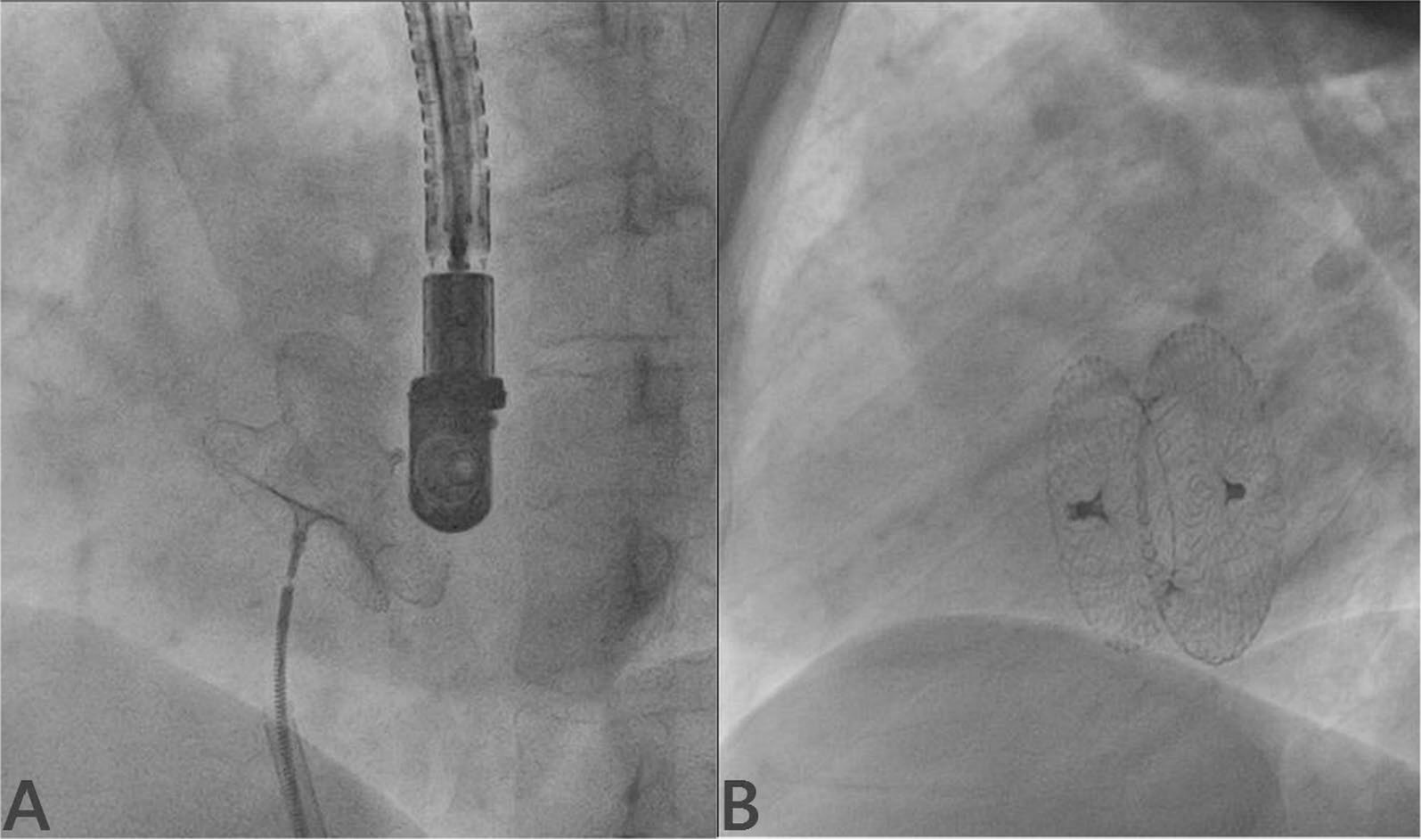
Device position on LAO view prior (**A**) and post-release (**B**)

In patients with absent or inadequate aortic rim, device was deployed using right upper pulmonary vein (RUPV) technique (Figs. 5, 6). In this method, the wire was placed in the right upper pulmonary vein and other maneuvers were kept same. The left disk was partly deployed in RUPV, and the sheath was quickly pulled to open its remaining part which led to jumping of left disk, thus aligning it parallel to septum [8]. Waist and right disk were quickly deployed before prolapsing of left disk. Once properly deployed, the rest of maneuvers were same.

**Fig. 7:**
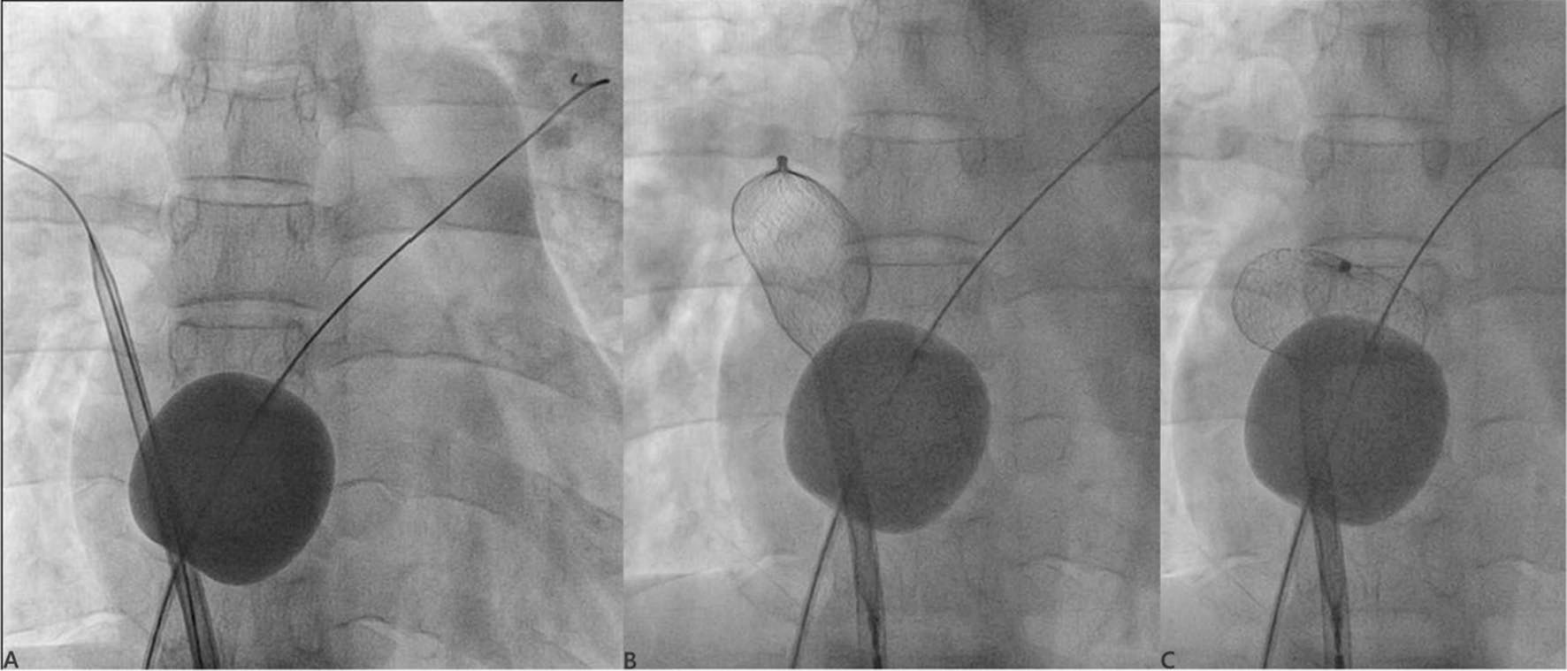
Device deployment using BAT technique. (**A** Equalizer balloon was inflated in RA touching the septum over the wire which was parked in LUPV, while the sheath was placed over the wire parked in RUPV; **B** left disk was partially opened just outside RUPV; **C** sheath was quickly pulled to open its remaining part)

**Fig. 8:**
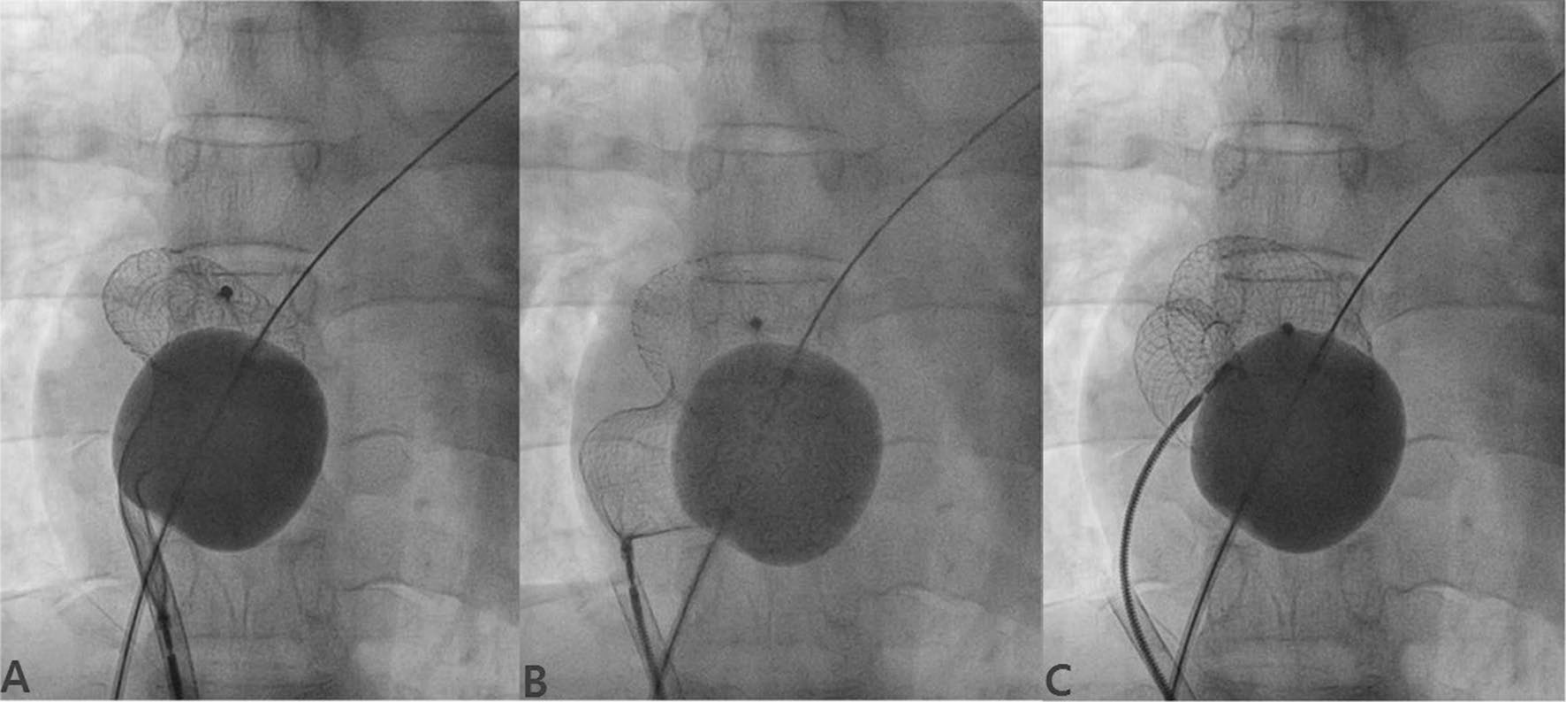
Device deployment using BAT technique. (**A** Waist and right disk are quickly deployed after retracting the sheath into RA; **B** properly opened disks; **C** Minnesota maneuver was performed to check the stability of the device)

In cases of large defect where the chance of prolapse of left disk into RA was high, balloon-assisted technique (BAT) was used. Here, the wire is placed into RUPV and LUPV from the corresponding femoral venous access. Equalizer balloon (EB) (Boston Scientific, USA) was positioned into RA over wire which was parked into LUPV (Fig. 7A). This balloon comes in various sizes (20, 27, 33, 40 mm) and should be larger than the size of defect [9]. It prevents the prolapse of left disk into RA when inflated. Once the device is pushed till the tip of sheath positioned into RUPV, the equalizer balloon was inflated and pushed over Amplatz wire till it touches the septum which was confirmed on TTE/TEE. Left disk was delivered just outside RUPV by little retracting the sheath. Keeping EB still inflated, rest of device (waist and right disk) was delivered in usual fashion (Fig. 7B, C) where both left and right disk appeared to be separated and assumed a dumbbell shape (Fig. 8). After confirming the apposition of left disk, balloon was deflated which helped in flattened out of both disks to align themselves with interatrial septum. Once properly apposed, deflated balloon was gradually withdrawn and the device was finally released in usual fashion.

**Fig. 9:**
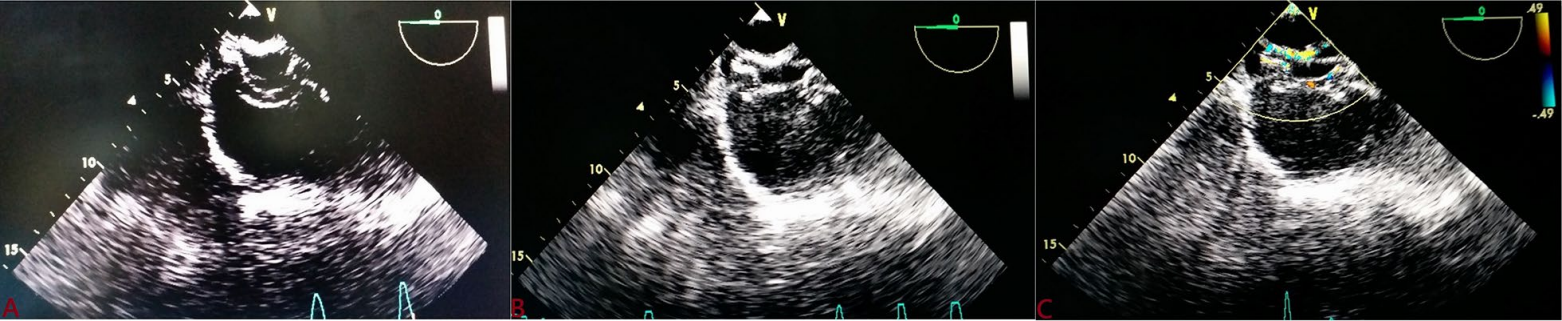
TEE showing proper placement of device with disks catching the rim properly (**A**;**B**) and post-deployment residual shunts (**B**)

In all cases, device position was confirmed (Fig. 9) and post-deployment residual shunts were classified as minimal to severe [10]. All patients were loaded with aspirin (3–5 mg/kg) preprocedure and continued till 6 months. Follow-up TTE was performed on the following day, 1 month, 6 month, and repeated every 6 months to assess device position and erosion.

### Data collection and follow-up

Efficacy was defined as successful closure of defect without significant shunt (not more than mild) on 6 months of follow-up, whereas safety was defined as successful deployment without either major complications (death, device embolization, cardiac perforation, pericardial effusion leading to tamponade, stroke, infective endocarditis, complete atrioventricular block) or late embolization, erosion, and residual shunt on follow-up.

### Statistical analysis

Statistical analysis was performed using Statistical Package for Social Sciences (SPSS; Chicago, IL, USA) program, version 20. Categorical variables were expressed as frequency and percentages, while continuous variables were expressed as mean ± standard deviation (SD).

## Results

Baseline characteristics and clinical presentation are shown in Table 1. A total of 320 patients (238 females, 82 males) were enrolled. The mean age and weight were 14.6 ± 3.1 (range 6–29 years) and 30.2 ± 5.4 (range 10-53 kg), respectively. The mean diameter of ASD and device was 21.4 ± 5 mm (range 12–36 mm) and 26.9 ± 4.2 mm (range 14–40 mm), respectively. The commonest indication for device closure was right ventricular overload.

**Table 1:** Baseline demographic and clinical presentation of patients (*N* = 320)

Characteristics	No. (%)
Sex (female/male)	238(74%)/82(26%)
Age (years)	14.6 ± 3.1(6–29)
Clinical indications	
a. Right ventricular overload	320(100%)
b. Failure to thrive	81(25.3%)
c. Recurrent respiratory tract infection	73(22.8%)
d. Exercise intolerance	57(17.8%)
e. Pulmonary hypertension	13(4.1%)
f. Palpitation	9(2.8%)
Rhythm	
a. Sinus rhythm	313(97.8%)
b. Atrial fibrillation (AFib)	5(1.5%)
Deficient rims (< 5 mm)	52(16.2%)
a. Posterior	14(4.3%)
b. Aortic	33(10.3%)
c. Superior	5(1.2%)
d. Inferior	0(0)
Absent aortic rim	11(3.4%)
Weight (kg)	30.2 ± 5.4(10–53)
Pulmonary vs. systemic flow (Qp/Qs)	2.6(1.4–3.6)
Associated disease	7(2.1%)
a. Rheumatic mitral stenosis	2(0.6%)
b. Patent ductus arteriosus (PDA)	3(0.9)
c. Ventricular septal defect (VSD)	2(0.6%)
Normal situs solitus	317(99%)
Situs inversus	3(1%)

(*n* = 320; 100%) and failure to thrive (*n* = 81; 25.3%), respectively. Deficient rim (< 5 mm) either in isolation or in combination was observed among 52 (16.2%) patients which was most commonly attributed to aortic rim (*n* = 33; 10.3%), whereas aortic rim was completely absent in 11 (3.4%) patients. Two patients had Lutembacher syndrome and had undergone balloon mitral valvuloplasty before device closure. Three (0.9) and 2 (0.6%) patients had additional PDA and VSD which were also dealt percutaneously. In total, 314 (98.12%) patients out of 320 were discharged from hospital with device in-situ. The overall safety and efficacy was 97.5% each, as given in Table 2. TTE- and TEE-assisted device closure was performed in 298 (93.1%) and 22 (6.9%) patients, respectively. Most of the smaller defects requiring smaller device were implanted using conventional LUPV technique (*n* = 234; 73.1%), while relatively larger defects were deployed using RUPV technique (*n* = 42;13.1%) and Greek maneuver (*n* = 35; 10.9%). BAT was used exclusively for very large defect (> 34 mm) and/or deficient aortic or posterior rim in 9 (2.8%) patients. Three hundred and one (94.1%) achieved complete closure of defect immediately following the procedure, while 19 (5.9%) had some shunt following implantation of device. At 6 months of follow-up, only 2 patients had moderate residual shunt.

**Table 2:** Procedure characteristics and outcome of transcatheter closure of atrial septal defect (*N* = 320)

Variable	*N*(%)
Efficacy	312(97.5%)
Safety	312(97.5%)
Transthoracic guidance (TTE)	298(93.1%)
Transesophageal guidance (TEE)	22(6.9%)
Mean diameter of ASD (mm)	21.4 ± 5(12–36)
Mean diameter of the device (mm)	26.9 ± 4.2(14–40)
Balloon Sizing	26(8.1%)
Size difference	5.5 ± 2.6
Technique of deployment	
a. LUPV technique	234(73.1%)
b. RUPV technique	42(13.1%)
c. Balloon-assisted technique	9(2.8%)
d. Greek maneuver	35(10.9%)
Procedural time (mins)	23.5 ± 9.2(20—58)
Fluoroscopy time (mins)	6.8 ± 10.4(3.5–21)
Periprocedural complications	27(8.4%)
a. Cardiac death	0(0)
b. Device embolization	2(0.6%)
c. Cardiac perforation (CP)	1(0.3%)
d. Pericardial effusion (PE)	3(0.9%)
e. Transient supraventricular arrhythmias	15(4.6%)
f. Transient atrioventricular block	6(1.8%)
g. Local site hematoma	0(0)
i. Stroke	0(0)
Transient headache	8(2.5%)
Follow-up	
a. Late embolization	0(0)
b. Erosion	0(0)
c. Residual shunting at 6 months of follow-up	2(0.6%)
d. Nickel allergy	0(0)
Follow-up duration (months)	50.92 (12.5–89)
Hospital stay (hours)	29.4

Periprocedural complications were observed in 27 (8.4%) patients which were attributed to supraventricular arrhythmias (*n* = 15; 4.6%), transient atrioventricular block (AVB: *n* = 6; 1.8%), device embolization and pericardial effusion in 2(0.6%) and 3(0.9%) patients respectively while cardiac perforation was reported in 1 (0.3%) patient. All supraventricular arrhythmias were transient as a result of manipulation of hardware. Most of them reverted to sinus rhythm spontaneously while 3(0.9%) patients required diltiazem. In 3(0.9%) patients complete AV block was noted following deployment of large device after multiple attempts, out of which 2 (0.6%) patients had right bundle branch block at baseline. Device was recaptured and patients were referred for surgical correction. Remaining 3 (0.9%) patients had first-degree AV block which got normalized, after administration of intravenous dexamethasone, on the fourth day following device implantation.

**Fig. 10:**
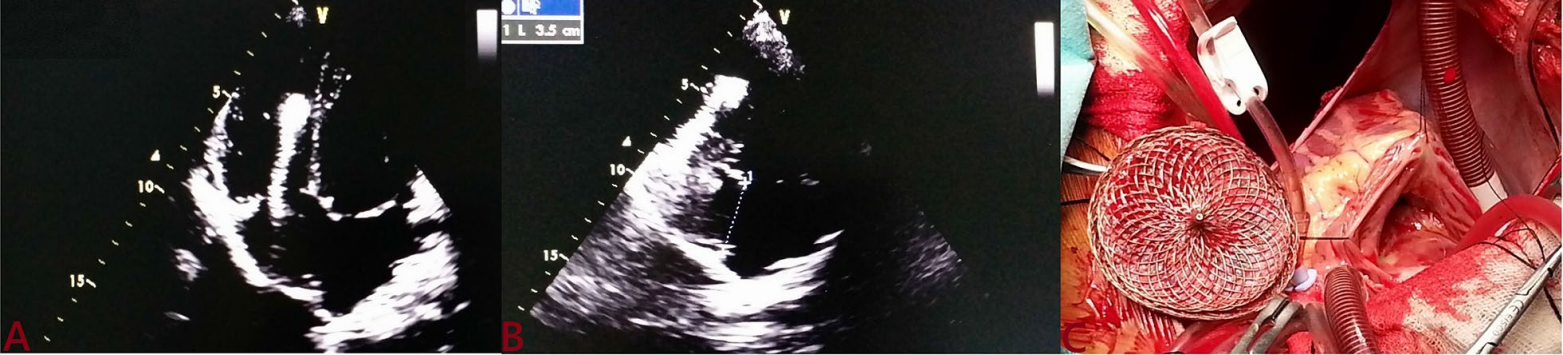
Dislodged device into right ventricle (**A**) in a patient with very large defect (**B**) who underwent surgery (**C** dislodged device)

Device dislodgement was reported in 2 (0.6%) patients, who got migrated to right ventricle on next day. Both were 40-mm device used to close large defect (36 mm) and had inadequate aortic and posterior rim. Both were surgically removed (Fig. 10).

Manipulation of large 14F sheath in a 16-year-old girl resulted in perforation of RA leading to cardiac tamponade. Pericardiocentesis was performed and emergent repair of defect followed by patch closure was performed. It resulted as device could not be deployed and sheath was mobilized from RA to LA leading to this complication. The mean fluoroscopy and procedural time were 6.8 ± 10.4 (range 3.5–21 min) and 23.5 ± 9.2 (range 20–58 min) respectively. Follow-up was performed using TTE with color Doppler on scheduled visit which showed no late embolization and erosion. Moderate residual shunt was seen in 2 patients at 6 months of follow-up. Nickel allergy was not reported in any of our patient [11, 12]. Five (1.5%) patients who had atrial fibrillation (AF) at baseline remained in AF. No neurologic events occurred except self-limiting headache (*n* = 8; 2.5%).

### Discussion

The key findings in our study were 97.5% safety and efficacy of transcatheter closure of secundum atrial septal defect using Cocoon septal occluder among diverse patient population (adult as well as pediatric) for small-to-large defects (> 25 mm) with no late events on mean follow-up of 4.5 years. Transcatheter closure has certain inherent advantages over surgery as it is minimally invasive, hospital stay is short, and it is associated with low morbidity and mortality.

Though this study had a single arm, the results of this study are comparable to those done for other devices and in a way reflect non-inferiority of this device. Efficacy using CSO in our study (97.5%) was similar to current-generation devices like ASO (97.6%), Gore Helex septal occluder (96.4%)[13], Cera septal occluder (99.7%), Biostar (97%), and Figulla Flexible Occlutech septal occluder (83%) [14].

Although TEE provides better clearer delineation and sizing of ASD compared to TTE, device placement can be performed using TTE which prevents introduction of esophageal probe and conscious sedation. In our study, TEE was used only in 22 (6.9%) patients. TCC of ASD using ASO under TTE has been demonstrated to be safe by Pan et al. [15] and Li et al. [16]. Good acoustic window is essential to adequately image the atrial septum.

Large defect defined as balloon-stretched diameter ≥ 34 mm in adults or echocardiographic diameter > 15 mm/m^2^ in children accounts for almost 20% cases. These defects are difficult to be dealt with conventional deployment technique as device do not orient parallel to IAS and tend to prolapse into RA. 24% of patients in our study had large ASD which was dealt using RUPV technique and Greek maneuver. Thanopoulos et al. [17] demonstrated 95% success in ASD closure in patients with large ASD with deficient aortic/posterior rim. BAT was used for device deployment for large defects in only 2.8% patients in our study in lieu of failure of Greek maneuver. As Cocoon septal occluder is softest and lightest among currently available devices [18], even the very large devices were successfully deployed without balloon assistance. Pillai et al. [19] reported 91% success with BAT among a series of 36 patients who had large defect (> 35 mm). Dalvi et al. [7] reported similar result in 14 patients with 100% success rate using BAT where the average ASD size was 32 mm. It has been noted that BAT improved success rate to 92% for large ASD compared to 16% using conventional technique [19]. In our case, BAT was used in 11 patients with success rate of 81.81%.

Major adverse cardiac events in our study (8.4%) were similar as reported with Amplatz device (7.2%) [20]. Device embolization was noted in 2(0.6%) patients in our study where as its reported incidence is 0.4–1.1% [21]. In both patients, defect was very large (36 mm) with absent aortic rim which was attempted with 40 mm using BAT. Initial deployment was successful but both migrated to right ventricle within 24 h (Fig. 10). As devices were very large, both patients were referred to surgery for removal as well as patch closure of defect. Most of embolization are acute (< 24 h) though late events have been reported as well. The risk factors for embolization are larger defect, larger device, undersize device, too small left atrium, deficient inferior/aortic rim, and flimsy rim [22]. Beside surgery, it can be retrieved percutaneously using snare. In our study, there was no episode of late embolization though it has been reported by Chess et al. in 2 patients [23], and Verma et al. in one patient [24].

Reported incidence of AV block (AVB) is 1% to 6% with all types of devices in literature [25] and was observed in 1.8% patients in our study. All episodes were transient in our study which was similar to findings reported by Chan KC et al. [26]. Irreversible third-degree AVB requiring pacemaker implantation has been reported as well [25]. Probable etiologies are inflammation and edematous compression of AV as a result of mechanical irritation by atrial disk, scarring of AV node and rarely vascular compromise of AV node [27]. The various risk factors are inadequate postero-inferior rims, greater device/height ratio, oversize device in small children, baseline conduction defect and weight < 15 kg. In our study, 3 (0.9%) patients developed complete AV block following deployment of large device, among these 2 patients, 13 and 16 years old, had a defect of 28 mm and had right bundle branch block at baseline. Third patient was a 7-year-old girl whose defect size was 22 mm with inadequate posterior rim requiring device of 24 mm. Her baseline electrocardiogram was normal. They developed third-degree AVB after device deployment. Considering the need of pacemaker in future, device was recaptured and they were referred for surgical correction. In remaining 3 cases, only first-degree AVB was noted which regressed with dexamethasone which was concordant with findings reported by Sudha et al. [28] which indicated that transient mechanical compression of AVN was likely reason.

Cardiac erosion leading to perforation is one of the most dreaded complications. There were no device related erosion in our study, while reported incidence varies from 0.1 to 0.4% [29]. In one case, immediate perforation of RA due to vigorous manipulation of 14Fr sheath in order to close 28 mm defect with 32 mm device was noted. We would like to emphasize that once sheath is into RA, it should be re-advance over diagnostic catheter (JR/MP) using floppy wire rather than blind manipulation. Device erosion can be acute as well as delayed (weeks to years). Though most of erosion has been reported with ASO, it has been observed with all types of devices. Divekar et al. [29] reported 24 cases of CE (both acute and delayed) which was responsible for neurological impairment and death in 3 patients each. In the largest series of 4,008 patients receiving CSO as reported by Thanopoulos et al. [30] over mean follow-up of 43 months, no case of erosion was reported and our finding was concordant with it. The observed factors which increases propensity of CE are inadequate (absent/deficient) aortic and/or superior rim, too much protrusion into the atrial or aortic wall, device straddling of aorta, older age, relatively stiffer occluder (ASO, Occlutech and Cardiac devices), oversized device, and multiple attempts at deployment. No case of CE by CSO may be attributed to its softness because of wire net and smooth surface as a result of nanoplatinum coating.

Ries et al. and Burian et al. described the significant increase in serum nickel levels after transcatheter closure of ASDs with ASO [31, 32]. Systemic allergic reaction to nitinol-containing device implanted for ASDand PFO closure have been reported [33, 34]. Nickel allergy manifest in form of migraine like headache, chest tightness, breathlessness, rash/urticaria, fever, and pericardial effusion [35]. Nickel allergy as a result of immunoallergic hypersensitivity reaction to nickel, was not observed in our study although headache was observed in 8(2.5%) patients which resolved spontaneously. The platinum-coating layer in CDO creates a biocompatible and noncorrosive zone, which can prevent the adverse effects from nickel release. No allergic reaction was reported in 15 patients after CSO implantation who had already tested positive to nickel hypersensitivity by Thanopoulos et al. [30]. No mortality attributable to the procedure occurred in our study.

## Conclusions

Percutaneous transcatheter closure of ASD by Cocoon septal occluder (up to 36 mm) is safe and feasible with high success rate and without any significant device-related major complications over long-term follow-up. With unique device design and excellent long-term safety, it could be preferred dual-disk occluder for transcatheter closure of atrial septal defect.

In most of the patients, ASD device can be safely deployed under transthoracic echocardiographic guidance.

## Limitation

Our study was single arm, lacking any comparison with other contemporary devices. Furthermore, complex ASD like multiple and aneurysmal were not included.

## Abbreviations

ASD: Atrial septal defect
TCC: Transcatheter closure
ASO: Amplatzer septal occluder
CSO: Cocoon septal occluder
RV: Right ventricle
TTE: Transthoracic echocardiography
TEE: Transesophageal echocardiography
IVC: Inferior vena cava
SB: Sizing balloon
LUPV: Left upper pulmonary vein
RUPV: Right upper pulmonary vein
MPA: Multipurpose catheter
BAT: Balloon-assisted technique
LAO: Left anterior oblique
